# Developments in Treatment Methodologies Using Dendrimers for Infectious Diseases

**DOI:** 10.3390/molecules26113304

**Published:** 2021-05-31

**Authors:** Nina Filipczak, Satya Siva Kishan Yalamarty, Xiang Li, Farzana Parveen, Vladimir Torchilin

**Affiliations:** 1 Center for Pharmaceutical Biotechnology and Nanomedicine, Northeastern University, Boston, MA 02115, USA; nin.filipczak@northeastern.edu (N.F.); yalamarty.s@northeastern.edu (S.S.K.Y.); xiang.li@jxutcm.edu.cn (X.L.); f.parveen@northeastern.edu (F.P.); 2 State Key Laboratory of Innovative Drug and Efficient Energy-Saving Pharmaceutical Equipment, Jiangxi University of Chinese Medicine, Nanchang 330006, China; 3 The Department of Pharmaceutics, Faculty of Pharmacy, The Islamia University of Bahawalpur, Bahawalpur 63100, Pakistan; 4 Department of Oncology, Radiotherapy and Plastic Surgery, I.M. Sechenov First Moscow State Medical University (Sechenov University), 119991 Moscow, Russia

**Keywords:** dendrimers, drug delivery, nanoparticles, antimicrobial therapy, antiviral therapy, infectious diseases, diagnostics

## Abstract

Dendrimers comprise a specific group of macromolecules, which combine structural properties of both single molecules and long expanded polymers. The three-dimensional form of dendrimers and the extensive possibilities for use of additional substrates for their construction creates a multivalent potential and a wide possibility for medical, diagnostic and environmental purposes. Depending on their composition and structure, dendrimers have been of interest in many fields of science, ranging from chemistry, biotechnology to biochemical applications. These compounds have found wide application from the production of catalysts for their use as antibacterial, antifungal and antiviral agents. Of particular interest are peptide dendrimers as a medium for transport of therapeutic substances: synthetic vaccines against parasites, bacteria and viruses, contrast agents used in MRI, antibodies and genetic material. This review focuses on the description of the current classes of dendrimers, the methodology for their synthesis and briefly drawbacks of their properties and their use as potential therapies against infectious diseases.

## 1. Introduction

Nanotechnology comprises a multidisciplinary approach at the nanometric level for characterization, manipulation and arrangement of molecules in a systematic way. It utilizes the diverse knowledge, concepts and novel ideas from different fields of sciences including engineering, physics and biological chemistry to design materials having 1–100 nm size range. The pharmaceutical nanotechnology aims to deliver the therapeutic moieties specifically at the site of disease to reduce unwanted and toxic effects of drugs to normal cells of organisms. The nanocarriers can alter the innate physicochemical, biological and pharmacokinetic properties of drug molecules for better therapeutic outcomes [1,2,3].

The recent advancements and innovations made in the field of molecular and polymer chemistry have led to a new domain called dendrimer chemistry. Dendritic nanoparticles have gained a lot of attention and interest from various research groups for biomedical and pharmaceutical applications. The word dendrimer originated from the Greek word Dendron meaning tree and the idea to synthesize such macromolecular, hyperbranched architectures originated from the numerous patterns observed in nature. The vascular system, tree branches, neuronal system, light patterns, tributary origination, explosions or erosions are a few examples found in nature [4,5].

The first attempts to characterize the architecture of dendrimers with a unique four-dimensional, core–shell with alterable surface groups were initiated by two research groups in 1978 and 1985 (Buhleier and Tomalia respectively). The dendrimers have multiple trees like branches originating from a central core molecule with various terminal functional groups giving them a unique and dense structure with more surface area for reaction. The size of dendrimer is optimized so that it can take benefit from enhanced permeability and retention effect leading to passive targeting. The control over synthesis steps of dendrimer result in unique biological properties by creation of various layers around a central core molecule. The efficient loading of therapeutics and imaging materials, specified desired delivery, versatile selection of administration route, monodispersed system, improved pharmacokinetic and pharmacodynamic profiling are some of the advantages of this unique drug delivery system [6]. These tree-like hyperbranched structures are also compatible with the various stimuli applied externally to control the release of drug such as temperature, pH, photons, magnetic fields and X-rays [7].

## 2. Types of Dendrimers

Based on the method of synthesis, physicochemical properties, physical structure and shape, the dendrimer chemistry has been described for several types.

### 2.1. PAMAM Dendrimers

Polyamidoamine (PAMAM, now commercially available as StarburstTM) has been extensively investigated since 1997 for their excellent solubilizing capacity, biocompatibility and comparatively low toxicity [8]. These dendrimers have been prepared using five different methods utilizing divergent, convergent, click chemistry, self-assembling, LEGO chemistry synthesis method with Michael addition and some amidation reactions [9].

The number of terminal primary amine groups, the size (<1.0–11.4 nm) and molecular weight of dendrimer increases with the generation number (0–9). The size of their cavities increases while polarity increases as generation proceeds. The shape and structure of dendrimer also becomes diverse from open and loose to compact and dense with increasing reaction steps. The toxicity and biocompatibility of the dendrimer is a major concern. The transport of drug molecule, the surface charge, generation size and concentration of a particular dendrimer play an important role in evaluation of the safety of PAMAM dendrimers. The presence of strong positive charge on the surfaces of PAMAM dendrimers limits their clinical applications due to rapid clearance from the systemic circulation and cytototoxicity. The higher generations of PAMAM dendrimers also involve high cost, time and labor consumption [10]. To address this issue, modification of structures through various strategies such as PEGylation, acetylation, conjugation and half generation dendrimer synthesis with carboxylate terminal groups had been explored [11]. Modification of structure is required to resolve toxicity issues. The presence of anionic or neutral surface groups significantly reduces the toxicity due to shielding of positive charge. PAMAM succinamic acid dendrimers, synthesized with the reaction of succinic anhydride molecules with amine-terminated PAMAM dendrimers, are full generation dendrimers with less polydispersity compared to half generation carboxylate terminated dendrimers. These types of dendrimers can be better candidates for the transport of pharmacologically active guest molecules as compared to standard half generations [12].

### 2.2. PPI Dendrimers

Polypropylene imine dendrimers are the oldest dendrimers first synthesized by Voegtle et al., 1978 [13]. The divergent method finds its implication with diamino butane (DAB) or EDA as core. The surface of PPI dendrimers contains cationic groups that facilitate the interaction between their positive charge and membrane negative charge [14]. Currently up to five generations of PPI dendrimers have been prepared for applications in drug delivery and a theranostic [15].

### 2.3. PAMAM Core–Shell Tecto Dendrimers

Since the discovery of the family of marketed PAMAM dendrimers, various attempts have been made to modify the physicochemical and biological properties of dendrimers. Discovery of several defects of single generation dendrimers such as limited drug loading capacity or gene transfection efficiency of lower generation dendrimers, and complexity, cost and time consuming synthesis and toxicities related to higher generations led to search for more effective alternatives to overcome these limitations. The higher generations using as core and lower generations as a shell surface covalently linked to a core led to the synthesis of a new type of dendrimer with advantages over conventional dendrimers [16]. The formation of covalent bonds and supramolecular host-guest assembly methods have been proposed for the synthesis of these novel architectures. Shi and co-workers recently reported the successful synthesis of CSTDs using a host–guest assembly method. They attached β-cyclodextrins to G5 PAMAM amine-terminated dendrimers as a core and adamantane or benzimidazole modified G3 dendrimers as shells that can be a promising vehicle for gene transfection applications. However, further in depth investigations are ongoing to determine the physicochemical characteristics, biological applications and limitations of these supramolecular constructs [17,18,19].

### 2.4. Chiral Dendrimers

Chirality is an important phenomenon during various biological reactions and recognition processes. The racemic active ingredients require enantioselective synthesis methods or direct chiral separations to resolve the enantiomers that are pharmacologically active from the rest that may be inactive or even toxic. The chiral dendrimers serve as chiral selectors during electrokinetic chromatographic techniques to separate the optically active compounds for analysis or synthesis purposes [20]. The salient features and control over the synthesis of dendrimers allow introduction of the chirality through various methods. The presence of stereogenic centers in the core, on the branches or surfaces of dendrimers make them optically active. As the name indicates, these types of dendrimers possess branches that are chemical analogues with a chiral core. The chiral resolutions and analysis time could be regulated by critical control over size and concentration of chiral dendrimers utilized [21,22]. These types of dendrimers are being extensively investigated as chiral selectors in modern supercritical fluid chromatographic systems that are relatively newer and promising in terms of ease, economics and speed of analysis [23]. The highly selective biological actions of chiral molecules due to specific enzymatic associations offer countless applications in drug delivery systems. Thirunarayanan et al. synthesized chiral core-based triazole dendrimers through a click chemistry approach for specific antibacterial properties to overcome resistance [24].

### 2.5. Frechet-Type Dendrimers

These were synthesized by Hawker and Frechet [25]. They contain poly-benzyl ether as a hyperbranched skeleton with carboxylic groups as terminal groups that facilitate further reactions.

### 2.6. Liquid Crystalline Dendrimers

A new class of dendrimer with a liquid crystal nature organized to form lamellar or globular architecture has been widely explored. The calamitic (rod-like) and discotic (disc-like) molecules form a skeleton called mesophage, which is liquid crystalline in nature [26,27]. The mesogenic liquid crystalline type of monomers facilitate formation of an interfacial layer. This liquid crystalline alignment can be spatially controlled by irradiation with light of different wavelengths [28].

### 2.7. Peptide Dendrimers

These dendrimers are radial or wedge type in shape with a peptide core that is synthesized frequently by both convergent and divergent methods. These dendrimers have peptide bonds in their structure and are formed by polymerization of amino acids. There are three types Type I, Type II (covalent PD) and Type III (non-covalent PD) depending on the location of amino acids [29]. These types of dendrimers have been extensively utilized as surface active agents and gene and drug carriers. Several reports supporting extended therapeutic activity of loaded therapeutics have been published recently [30,31].

### 2.8. Hybrid Dendrimers

Linear types of polymers combine with dendritic types to form a dense, compact and globular structure, The dendrimers are combined with other drug delivery systems to form novel constructs with superior characteristics [32]. Albumin nanoparticles stabilized by dendrimers were presented as a unique platform for sustained release of therapeutic moieties, genes, siRNA and miRNA. Supramolecular nano constructs were established with electrostatic controlled gelation of G4 PAMAM dendrimer by encapsulation of paclitaxel [33].

## 3. Synthesis Routes

Three unique domains of dendrimers include core, various repetitive branches and terminal groups have been prepared with specific and unique physicochemical and biological properties intended for various diagnostic and therapeutic purposes [34]. Different generations of dendrimer exist depending upon the number of branches that extend from a central core to outside terminal groups with increasing the molecular weight of dendrimers.

### 3.1. Divergent Method

This is a traditional synthesis method first introduced by Tomalia et al. in 1985 [35]. The first report of the divergent method was published with PAMAM dendrimer generations of 1–7 with molecular weights up to 50,000 Da [36]. This synthesis route begins from a core scaffold and involves attachment of monomer units in a sequential manner around core. The extensive utilization of commercial reagents at each repetitive step increases the mass of the end-product due to generational growth. Each new layer of the monomer gives rise to a new generation of dendrimer with a terminal surface that can be modified for a specific desired function. Each generation must be fully accomplished to minimize branching defects associated with incomplete functionalization or side reactions [37]. This method is very quick and efficient for syntheses of high molecular weight up to generation 10 or even more and essentially monodispersed dendrimers. The generational defects due to structural crowding, incomplete Michael addition, retro-Michael reactions or intramolecular cyclization are inevitable. The subsequent purification to yield defect-free dendrimers by methods such as dialysis, precipitation or combination of two or more methods is a technical challenge for successful preclinical trials. The commercially available PAMAM, PPI and phosphorus-based dendrimers are synthesized by this method [38].

### 3.2. Convergent Method

**Figure 1:**
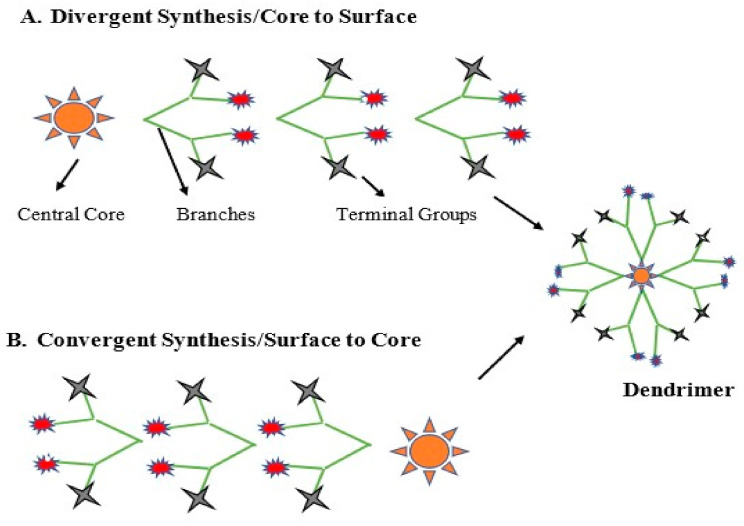
The construction of dendrimers: two main architectural methods designs.

This method was introduced by Hawker and Frechet [39]. The first dendrimer produced by this method belonged to the polyarylether dendrimer family [40]. This is a reverse method in which the synthesis starts from the surface groups and ends at the inner core. The complete control over the structural integrity of dendrimer is possible by this process to produce designer type dendrimers for specific applications. The size and shape of dendrimer is tunable with the selection of appropriate dendrons that might also be expensive. The multiple branches of dendrimers are produced first and are attached to a core molecule once they reach the desired generation number [41]. This method avoids branching defects and minimizes purification procedures since the impurities produced are relatively different in molecular weight. The synthesis of higher generation dendrimers is less successful due to increased steric hindrance as the number of monomer layers increases [38]. Bondareva et al. utilized both convergent and divergent approaches (Figure 1) in their study to evaluate the pros and cons of the two methods. They described the synthesis of first-to-third generations of sulfonamide-based dendrons with reactive chlorosulfo-groups at their focal points. They concluded that the convergent approach is limited up to the third generation while the divergent approach can achieve higher generations [42].

### 3.3. Click Chemistry

This approach utilizes benefits of two traditional approaches and combines them to produce dendrimers with structural diversity. It is also called the double stage convergent or the combined divergent/convergent approach [43]. The first report to use this method was published in 2004 when triazole-based dendrimers were produced using click chemistry. This method was expected to be very efficient as the only byproduct was NaCl and all the dendrons were collected as pure solids. Hence this method did not require any purification step via chromatography, making it a robust method for industrial scaleup [44].

### 3.4. Generation of Dendrimers

**Figure 2:**
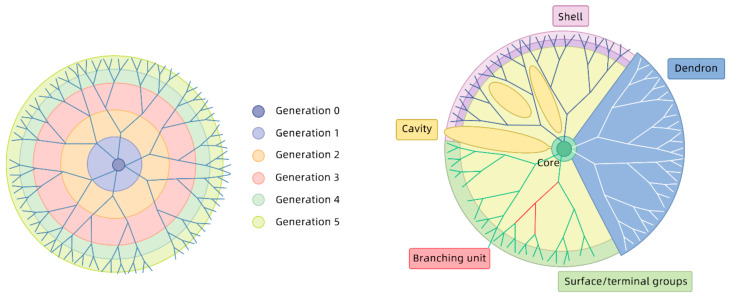
The typical structure of PAMAM dendrimers.

Dendrimers are a class of hyperbranched, monodisperse synthetic macromolecules with a well-defined core, internal space and surface functional groups. These macromolecules have a dendritic molecular structure, with a series of layered branches extending from the central core, hence the name dendrimer [45,46,47,48]. Poly(amidoamine) (PAMAM), for example, is a class of hyperbranched dendrimers synthesized through step-by-step repetitive reactions of branching units. The typical structure of PAMAM dendrimers (Figure 2) is comprised of three parts: (1) a core for initiation of branches; (2) a scaffold comprising repetitive units; (3) modifiable surface functional groups. Owing to their regular structure, precisely controllable molecular morphology and surface functional groups, PAMAM dendrimers are promising for a variety of biomedical applications. The molecular weight of dendrimers increases precisely with an increase in the number of generations. The molecular structure is spherical when the number of generations increases to 4. The surface functional groups of dendrimers can be modified and functionalized due to their unique structure. Thus, some types of dendrimers can be made soluble in water and linked to targeting and drug molecules for tumor-specific cell therapy, and gene therapy applications [49,50]. Metal nanoparticles (NPs) and drug molecules can be physically encapsulated in the internal cavities of PAMAM dendrimers. The resulting nanocomposite structure is stable and free of concentration [51,52]. Owing to their unique physiochemical properties, PAMAM dendrimers have become a popular carrier material with a wide application prospect in nanobiomedicine.

## 4. Application of Dendrimers in Treatment of Infectious Diseases

Due to their unique structural features, dendrimers are likely to be used in many fields of science and industry in the future. Much attention is now paid to research into the use of these polymers in medicine, chemistry, genetic engineering and environmental protection. The greatest interest of scientists has arisen from applications in medicine, which satisfy the need for better and more effective forms of therapy, especially in the case of diseases for which so far there is no cure. Trends in research on the use of dendrimers in medicine are divided. One uses these compounds as carriers for other substances, and the other is to conduct studies on the therapeutic properties of the nanoparticles themselves as shown in Table 1.

Until recently, infectious diseases caused by pathogenic microorganisms, including bacteria, viruses, parasites and fungi, were rationally controlled by a wide range of antimicrobials. However, widespread use antibiotics and antimicrobials led to the emergence of many new mutant and treatment-resistant strains [69]. There was no doubt that there was a need for new compounds with a broad spectrum of actions. Several research groups focused on dendrimers, which showed both antiviral and antimicrobial activity [70]. Discoveries made by Chen and Cooper [71], Mintzer and Grinstaff [72] and Balogha [73] showed that dendrimers could be very active against pathogens when appropriate modification of dendrimer’s surface was applied. Their broad activity may be due to bacterial membranes damage. Biofilm formation is inhibited and used to active compounds mimicking the action of detergents and antimicrobial peptides [74]. Antimicrobial dendrimers showing low cytotoxicity to eukaryotic cells are being investigated as new drugs for a variety infectious diseases, especially those which are highly lethal or incurable [75].

### 4.1. Dendrimers as Antiviral Agents

#### 4.1.1. Human Immunodeficiency Virus (HIV)

One of the infectious diseases for which no completely effective treatment has been found so far is AIDS caused by HIV. According to statistics from the United Nations Joint Program on HIV/AIDS (UNAIDS), approximately 37.9 million people worldwide have been infected with this virus. Currently a combination antiretroviral therapy (cART), with three or more drugs, is the most common and effective method of combating HIV infection. The downside of this therapy is the side effects associated with the administration of cART, including the risk of hyperlipidemia, adipose tissue redistribution and diabetes [76]. In addition, cART also weakens CD4 T cells, which are designed to kill HIV replicating cells [77]. Recently, these limitations of cART have been mitigated through the use of nanotechnology with targeted drug delivery and controlled drug release profiles in clinical trials. Currently, the most commonly used carriers for antiviral drugs are: dendrimers [78], nanosuspensions [79] and polymer micelles [80].

Currently, a preparation called VivaGel, based on the structure of dendrimers, produced by the Australian company Starpharma, has passed Phase III clinical trials and is in the process of obtaining FDA approval. This carbomer gel contains a fourth-generation poly-l-lysine dendrimer containing naphthalene disulfonate surface groups and a benzhydrylamine amide center (SPL7013). The strongly polyanionic surfaces of the dendrimers are believed to bind gp120 proteins on the surface of the virus. Via the gp120 proteins, the virus normally attaches to CD4 receptors on human cells to initiate the transfection process [78,81,82]. The VivaGel preparation has the ability to absorb HIV into the dendrimer, preventing it from spreading throughout the body [78]. Clinical trials have also confirmed the safety and efficacy of VivaGel^®^. Experimental results show the high effectiveness of this compound in blocking the transmission of HIV and herpes simplex virus (HSV), which makes it possible to fight AIDS.

**Figure 3:**
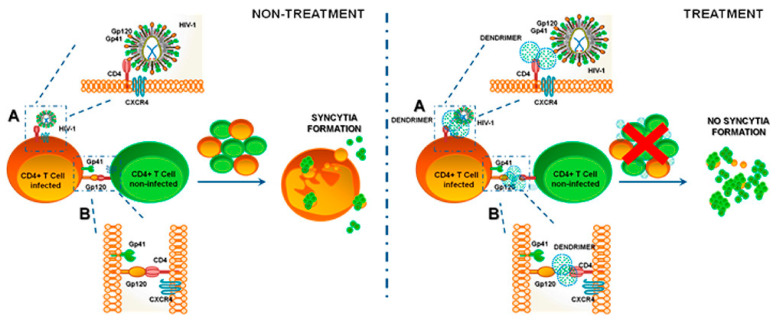
An example of anti-HIV activity of carbosilane dendrimers based on the gp120/CD4 receptor. Prevention of syncytia formation. Small T cell-formed syncytia can transfer virus to uninfected cells is another advantage of carbosilane dendrimers [83]. Reprinted from Guerrero-Beltran C. et al. *Anionic Carbosilane Dendrimers Destabilize the GP120-CD4 Complex Blocking HIV-1 Entry and Cell to Cell Fusion. Bioconjug. Chem.* 2018. Copyright © 2018, American Chemical Society.

The same antiviral mechanism that blocks the gp120/CD4 interaction (shown in Figure 3 [83]) is also utilized by the G2-S16 polyanionic carbosilane dendrimer with a silica core and 16 sulfonate end groups. In vivo results showed inhibition of HIV-1 transmission at an early stage of replication [84]. The G2-S16 dendrimer was also combined with the tenofovir reverse transcriptase (TFV) inhibitor or the CCR5 entry inhibitor maraviroc (MRV). The results obtained confirmed the synergistic effect of dendrimers and inhibitors, which confirmed that the developed combination is a good candidate as an antiviral agent for HIV prophylaxis, due to its stability at low pH [85,86].

SiRNA-based gene therapy is also used against HIV. In comparative studies, two second-generation carbosilane dendrimers (G2-NN16 and G2-03NN24) with the same quaternized amino terminal groups, but different core groups, were tested for their safety and efficiency in CD4 + siNef T cell transfection [87]. They reduced the expression of the helper Nef gene that enhances viral replication and spread by increasing viral titer. Although both dendrimers were able to transfect CD4 + T cells and enhance the inhibition of HIV-1 infection, they differed in efficacy. The G2-03NN24 dendrimer derived from the polyphenol core was stiffer, while the Si-core G2-NN16 was more flexible, resulting in increased cellular uptake by CD4 + T cells. As in PAMAM dendrimers, the efficiency of transfection increased with their flexibility [88].

In the development of anti-HIV therapy, PAMAM generation five dendrimers with a triethanolamine core and 96 amino terminal groups have also been used to provide siRNA combinations for CD4, TNPO3 and tat/rev proteins [89]. Temporary destruction of CD4 receptor blocks the fusion of HIV-1 and T cells. TNPO3 is a cellular factor that facilitates the transport of the cytoplasmic HIV-1 preintegration complex. Decreasing the level of this factor interferes with HIV-1 replication in the host cells. HIV tat/rev proteins are viral regulatory molecules that are essential in the HIV life cycle. PAMAM dendrimers were shown to systemically deliver a combination of functional siRNA. In vivo treatment of HIV-1 infected, viremic humanized mice provided effective protection against HIV-1 mediated T-cell loss with no apparent toxicity [90,91].

#### 4.1.2. Coronaviruses

**Figure 4:**
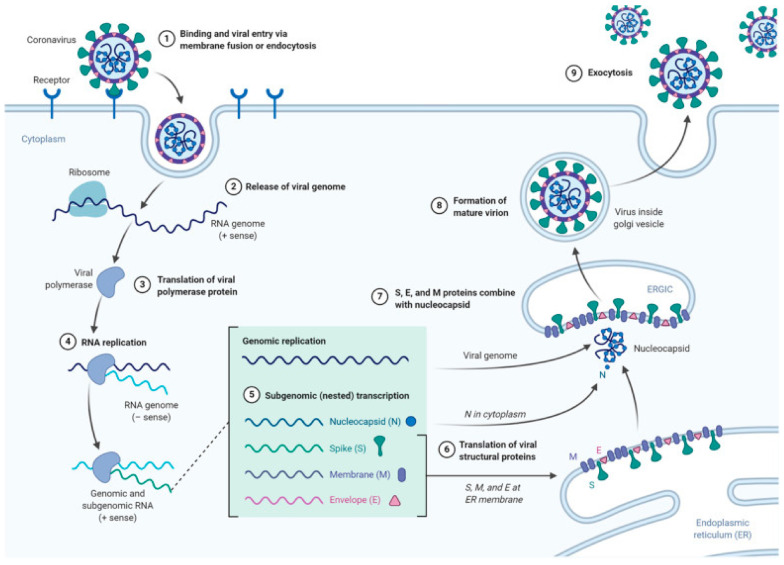
Life cycle of SARS-CoV-2. Virus entry through ACE2 receptor and mechanism of its replication in host cells [95].

Coronaviruses are a family of highly infectious viruses that cause severe respiratory disease, with a possible fatal outcome. These diseases originated in South America, West Africa, the Middle East and Asia and are currently spreading to other parts of the world. Currently a worldwide pandemic of coronavirus disease (COVID19) caused by severe acute respiratory syndrome coronavirus 2 (SARS-CoV-2) is affecting global health and the economy. Major symptoms of COVID-19, include acute respiratory disorder, excessive inflammation and an exaggerated immune response, which leads to a cytokine storm and progression to acute lung injury and often death. SARS-CoV-2 binds to human angiotensin-converting enzyme-2 (ACE2) receptors and induces pneumonia associated with hypercoagulability with hyperfibrinogenemia and large vessel thrombosis. SARS-CoV-2 is closely related to SARS-CoV, which appeared in the world in 2002 and 2003. SARS disease was recognized in about 8000 people with a mortality ratio about 10%. MERS disease has appeared sporadically in the Middle East since 2012 and it is believed to have caused 900 deaths [92,93]. SARS-CoV-2 and other coronaviruses are RNA viruses, which uses the host genome for the synthesis of viral genomic RNA and Mrna, which leads to the release of the new virions produced in the host cells [94] as shown on Figure 4.

The spike protein is most important for virus–cell receptor binding and virus–cell membrane fusion, which then becomes an effective target for CoV vaccine design. Two of the now currently available vaccines contain mRNA sequence of this protein [95] as an LNP-encapsulated mRNA (Moderna/NIAID) [96] or as a conjugate with the LNPs (BioNTech/Fosun Pharma/Pfizer) [97]. These types of vaccine may also potentially be delivered by dendrimers. Messenger RNA of the tensin homolog (PTEN) and phosphatase was successfully delivered by a modified PAMAM (generation 0) dendrimer coformulated with poly(lactic-co-glycolic acid) (PLGA) and ceramide-PEG in a polymer-lipid hybrid nanoparticle in vivo [98]. Additionally, dendrimer lipid nanoparticles (mDLNPs) comprised of 1,2-dimyristoyl-sn-glycerol-methoxypoly(ethylene glycol) 2000 (DMG-PEG), cholesterol, 5A2-SC8 dendrimer and DOPE were recently used to increase delivery of mRNA into liver tissue [99]. A similar formulation, built of dendrimer/DOPE/cholesterol/PBD-lipid/mRNA and theranostic mRNA was used to deliver to tumor tissue [100]. These recent findings suggest that dendrimers have potential to be mRNA vaccine delivery vehicles. Dendrimers as a potential vaccine carrier should provide protection against mRNA degradation by nucleases and shield its negative charge during circulation, and to facilitate crossing the cell membrane to reach the cytoplasm [101]. The advantage of using dendrimers as a vaccine against coronaviruses is that their structures provide high density of surface modifiable functional groups. They have low immunogenicity and a large number of positive charges on the surface that can be used to complex RNA or DNA [41,102]. On the other hand, enzymatic biodegradation of the dendrimers may be hindered due to steric factors and consequently lead to toxicity related to the accumulation of these materials in tissue [101].

Recently dendrimers were also used as the antiviral agents themselves against MERS-CoV. Antiviral activity of three types of dendrimers, including polyanionic dendrimers comprising the terminal groups sodium carboxylate (generations 1.5, 2.5, 3.5, and 4.5), hydroxyl (generations 2, 3, 4, and 5), and succinamic acid (generations 2, 3, 4, and 5) and polycationic dendrimers containing primary amine (generations 2, 3, 4, and 5) were investigated. All the dendrimers mixed with the MERS-CoV at a final concentration of 10 μM were able inhibit activity in plaque formation. These dendrimers proved to be a basis for further research as an antiviral therapy [103]. Recent studies also showed that topical application by inhalation of peptide dendrimer carrying SARS-CoV-2-specific modified siRNA can potentially be used for the treatment of SARS-CoV-2-induced lung inflammation [104].

#### 4.1.3. Ebola Virus

The Ebola virus belongs to the Filoviridae family and causes hemorrhagic fever. In Africa, where the virus most frequently appears, the disease it causes is characterized by a high mortality rate. At the moment, there is no vaccine against this virus and no effective treatment for its carriers [105]. The Ebola virus capsid consists of a trimer of a highly glycosylated glycoprotein that is recognized by both DC-SIGN and DC-SIGNR/L-SIGN type C lectins. These receptors are considered a potential gateway of infection for this virus, and hence, a strategy of blocking these receptors was used to block the entry of the Ebola virus [106,107]. The Boltron dendritic polymer used as a core when combined with 32 mannose groups on the surface, showed increased antiviral activity [108,109]. A modified multivalent version of this dendrimer showed increased antiviral activity in the nanomolar concentration range in a pseudotyped Ebola virus infection model. The results obtained indicate that the use of a glycosylated form of dendrimers may be a good strategy for the development of antiviral drugs [110].

Another strategy to fight Ebola virus is to prevent infection by vaccine usage. Recently ionizable dendrimer-based nanomaterial, a lipid-anchored PEG and self-replicating RNA were used together as a vaccine nanoparticles—MDNPs. The platform created was used to carry and successfully deliver RNA, which results in antibody production and antigen-specific CD8+ T-cell responses towards the encoded protein antigen. The platform can be also used as a vaccine against *Toxoplasma gondii* and H1N1 influenza [111]. For Ebola vaccine creation, PAMAM G4 dendrimers were also used. The dendrimers delivered artificial polyepitope T-cell immunogens in the form of a DNA. Due to relatively low immunogenicity, this approach needs more research [63].

#### 4.1.4. Influenza Virus

As mentioned previously dendrimers can be used as a vaccine carrier against H1N1 influenza. They are also used as a treatment carrier. For example, sialodendrimers can inhibit the process of hemagglutination of human erythrocytes induced by influenza virus. The first step in the infection of a cell by a virus is the attachment of the virion to the cell membrane. Adhesion occurs through the interaction of the hemagglutinin receptor on the viral surface with sialic acid groups on the cell surface [112]. Sialodendrimers bind to hemagglutinin, thereby preventing adhesion of viruses to cells. The attachment of a-sialic acid fragments to the surface of the dendrimer increases the therapeutic effect and allows the polymer molecule to achieve greater inhibitory activity during influenza infection. The effect increased the more sialic acid groups on the surface of the dendrimer molecule [113]. Recently PAMAM (G1) dendrimers conjugated with either 3′-sialyllactose (3SL) or 6′-sialyllactose (6SL) were synthesized as host-specific inhibitors of influenza virus infection and showed promising activity [114]. Carbosilane dendrimers with hemagglutinin binding peptide against influenza virus types H1N1 and H3N2 were also prepared and showed strong inhibitory activity against them [115]. A less effective strategy method use 4 sialic acid conjugated PAMAM dendrimers. They efficiently prevented the infection caused by influenza virus subtype H3N2, but not by the H2N2 subtype [116].

#### 4.1.5. Herpes Simplex Virus

In the case of herpes simplex virus, HSV, both polycationic (polyarginine and polylysine [117]) and polyanionic [118] dendrimers, have been used to prevent the virus from adsorbing to the cell surface. This phenomenon may result from the antagonistic interaction of the virus with the cell surface, which is a coupled interaction of the cell with the anionic structure of the receptor, or the interaction of the cationic virus with the cell. However, an advantage of polyanionic dendrimers over polycationic dendrimers is their lower cytotoxicity. In addition, peptide dendrimers and their derivatives (SB105 and SB105-A10) were also used. The dendrimers developed showed activity against HSV in an in vitro acid model. Dendrimer derivatives in combination with acyclovir showed a synergistic effect in vitro [119].

Another approach was to create poly (amide)-based dendrimers with a surface-attached gH (gH625) membrane peptide from herpes simplex virus type 1 (HSV-1) and surrounded by H glycoprotein. The developed dendrimer carriers showed antiviral activity and were non-toxic to cells in the range of concentrations used [120,121]. Polyanionic carbosilane dendrimers also showed activity against HIV-1 and HSV2 in the Vero cell model. Some of the dendrimers acted by direct binding to HSV-2, thereby inactivating, while others adhered to host cell surface proteins. As with peptide dendrimers, carbosilane dendrimers were synergistic with acyclovir and tenofovir. It has also been shown that topical vaginal or rectal administration of the formulation to BALB/c mice prevented the transmission of HSV-2 [122]. The mechanism of action of peptide-derivatized dendrimers, carbosilane dendrimers, galactose polysulfate-functional glycodendrimers, and PAMAM dendrimers used as germicides against sexually transmitted diseases is based on blocking a viral particle that binds to heparan sulfate on the cell surface or binds to cellular coreceptors [123].

#### 4.1.6. Other Viruses

Dendrimers are also used as a carriers or treatment itself for other viral infections. In Table 2 we summarized the other dendrimer applications for antiviral treatment.

### 4.2. Dendrimers as Antibacterial Agents

Unlike dendrimer, antiviral drugs, antimicrobial dendrimers contain cationic surfaces typically modified with amino groups or with tetraalkyl ammonium groups. Generally, these compounds adhere to the anionic cell wall of bacteria, causing damage followed by decomposition of the whole bacterium. An example of an antibacterial dendrimer is the PPI-based dendrimer modified with tertiary alkyl ammonium groups, which has been shown to be a potent antibacterial agent against both Gram positive and Gram negative bacteria [71].

#### 4.2.1. Gram Negative Bacteria

Dendrimer-glucosamine conjugate (PETIM-DG) created by Shaunak’s team was tested in a broad spectrum of infectious diarrheal diseases caused by *E. coli*, *Shigella* and *Salmonella*. The authors showed that the PETIM-DG conjugate was an inhibitor of the genus *Shigella*, inhibiting damage to the intestinal epithelial wall in rabbits. At the same time, it minimizes the invasion of bacteria and limits the expression of local cytokines [130,131].

Lysine-based dendrimers containing surface mannose molecules also showed high antimicrobial activity against *E. coli* strains [132]. Another group of dendrimers used against *E. coli* bacteria includes PAMAM dendrimers. They have been used to prevent premature labor due to *E. coli* infection in the guinea pig membranes and placenta models. The hydroxyl-terminated PAMAM dendrimers found their way into the cervix, thereby preventing *E. coli* from entering the uterus, reaching the fetus, and thus preventing premature labor [133]. Quaternary ammonium functionalized poly(propylene imine) dendrimers were also evaluated as a potential antimicrobial agents and showed promising results, although their function depends of the length of the hydrophobic chain [134]. It is well known that silver nanoparticles and silver complexes exhibit antimicrobial properties that last a long time. Due to the non-toxicity and good water solubility, silver complexes *P. aeruginosa* and *E. coli* [73].

Dendrimers can also be used as a preventive medicine for *Vibrio cholera*, another Gram-negative bacterium, which causes cholera. It is a life-threatening disease due to extensive loss of electrolytes. The main element that causes of this disease is protein AB5 secreted by the microorganism. The mechanism of action of this protein is to form the pore on the cell surface after recognition of GM1 ganglioside. Then the A subunit of the cholera toxin can block GTPase and consequently increases the amount of cAMP. Finally, water and ions are released from the cells [135]. 

One of the strategies used to prevent infection was with dendrimers with a core of 3,5-bis (2-aminoethoxy) benzoic acid and a GM1-mimic ligand [136] or with different types of lactose on the surface [137]. Unfortunately, both of the dendrimers were not investigated in the in vitro model. The affinity of dendrimers to cholera toxin was evaluated by ELISA and fluorescent spectroscopy. Another type of dendrimer that was used as an anticholera treatment included tetradendrimers (G1), okta (propylenoimin) (G2) dendrimers and Startburst^TM^ (PAMAM) G1 dendrimers functionalized with oligosaccharides. These dendrimers prevented B subunit of cholera toxin attachment to the cell surface [138].

Another approach used a dendrimer as a carrier for the known antimicrobial drug vancomycin. Serri et al. used G3 and G5 NH2-PAMAM dendrimers to encapsulate the vancomycin hydrochloride. As a result the delivery system reduced minimum inhibitory concentration MIC values by up to 64 times in *E. coli, K. pneumonia*, *S. typhimurium* and *P. aeruginosa* by increasing the permeation through the bacterial membrane [139].

#### 4.2.2. Gram Positive Bacteria

Among Gram-positive bacteria’s representatives, *Staphylococcus aureus* is one of the most resistant and most infectious. This opportunistic pathogen is especially dangerous for people with chronic conditions, a compromised immune system or people who have had surgery and those who used a catheter (e.g., dialysis patients) [140,141]. Thus, there is high need for a better treatment method. Usage of maltose-modified PPI G4 dendrimers against Gram-positive *S. aureus* provided efficient antibacterial activity and selectivity. At the same time, these nanoparticles showed little toxicity to eukaryotic cells [142]. Another type of G4 dendrimer modified with boronic acid and its antibacterial recognition properties were evaluated in *S. aureus.* B-PAMAM(G4) dendrimers selectively recognized the Gram-positive bacteria at neutral pH, which made them a useful tool for discrimination from Gram-positive bacteria [143].

**Figure 5:**
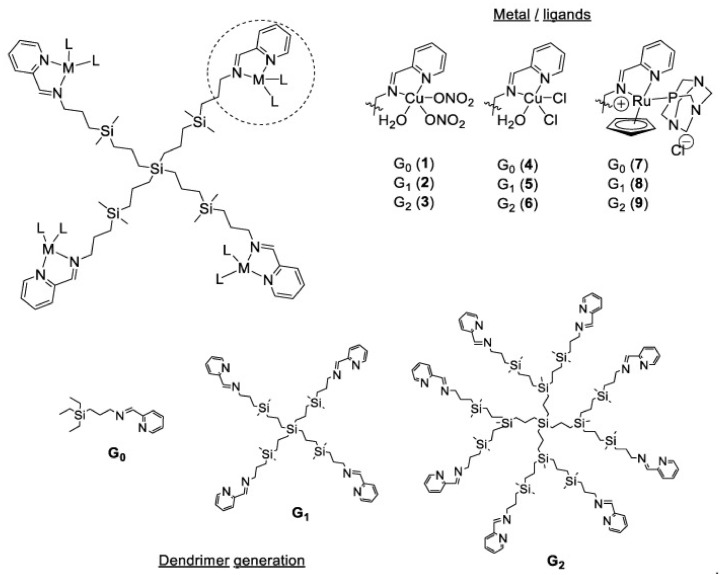
Chemical structure of carbosilane metallodendrimers [145].

The growth of *S. aureus* can be also inhibited by G1 polyphenolic carbosilane dendrimers functionalized with caffeic and gallic acids. The mechanism of action of these dendrimers is based on their antioxidant abilities, which corresponds to the number of hydroxyl groups in polyphenol structure [144]. Carbosilane dendrimers can be used as a scaffold for metal ions to help fight against Gram-positive bacteria as shown in Figure 5.

Copper (II) and ruthenium (II) in combination with G0, G1 and G2 carbosilane dendrimers were effective biocidal agents for *S. aureus* biofilms. It is desirable to inhibit bacteria’s growing as a biofilm since chronic and recurring infections are much related to bacteria’s ability to produce a biofilm structure [145], which make them resistant to the antibiotic’s treatment. Dendrimers can be used to increase the antimicrobial effect of the drug, as it happened in the case of DAB-core G0 PAMAM-dendrimer and ciprofloxacin conjugate. The observed synergistic effect of the dendrimer and ciprofloxacin conjugate is believed to be related to ciprofloxacin mode of action by primarily stabilizing the complex of topoisomerase IV, leading to DNA fragmentation in Gram-positive bacteria, since the control dendrimer did not demonstrate any antibacterial activity itself [146].

### 4.3. Dendrimers as Antiparasite Agents

#### 4.3.1. Malaria

Malaria is caused by the parasite of genus *Plasmodium*, which is a unicellular eukaryote carried by female *Anopheles* mosquitoes. Out of the five types of *Plasmodium* that infect humans *Plasmodium falciparum* is considered the most lethal one causing malaria. The parasite is transferred from person to person through blood transfusion, organ transplant, sharing of needles and also by the bite of a female *Anopheles* mosquito acting as a carrier [147]. The disease can be transferred from the mother to the child during childbirth. Tropical and the subtropical regions have the highest number of malaria cases in the world. Malaria has a huge impact, not only on the health, but also on the economy of a country thus leading to poverty [148]. Symptoms of the disease include, vomiting, headache and fever but severe cases may cause anemia, severe pains and coma [149].

Malaria is a debilitating and life-threatening disease. In 2019 nearly half of the world’s population was at the risk of its transmission. In 2019, 229 million cases of malaria were estimated worldwide. Children under the age of 5 were at the highest risk of malaria transmission as they were 67% of malaria deaths worldwide. Malaria cases (94%) worldwide come from the African and the sub-Saharan Africa according to the World Health Organization (WHO). Antimalarial drugs such as chloroquine, primaquine and artemisinin and its derivatives are used to treat malaria, but they produce severe toxicities and drug resistance [150]. Development of new delivery systems to treat malaria is needed. Dendrimers can act as an excellent delivery system to treat malaria due to its biocompatibility and biodegradability [151]. Derivates of dendrimers were synthesized by several research groups using 2,2-bis (hydroxymethyl) propionic acid (bis-MPA) and pluronic polymers, which contain chloroquine and primaquine. These polymers were investigated for their antimalarial activity by targeting *Plasmodium* infected RBCs (pRBCs) in both human (*Plasmodium falciparum*) and rodent (*Plasmodium yoelii*) and their in vitro results indicated a reduced IC_50_ od chloroquine and primaquine (3 and 4-fold respectively). These dendritic scaffolds have been shown to target pRBCs in comparison to normal RBCs. Amphiphilic bis-MPA derivatives have shown the ability to encapsulate antimalarial drugs due to their functional groups but also their ability to be cleaved by enzymes in the body to make them highly soluble in biological environments [152]. Recent approach to treat *Plasmodium falciparum* infections is to use artemisinin-derived dendrimers. These types of dendrimers can be considered as selective antimicrobial drug candidates, since they do not show cytotoxic effects in the healthy cells, but strong antimalarial activity [153].

#### 4.3.2. Leishmaniasis

Leishmaniasis is caused by the parasite *Leishmania,* which is transmitted by infected female sand fly [154]. Blood transfusion and sharing of needles may cause human to human transmission of the disease [155]. Both cutaneous and visceral forms of leishmaniasis exist. The symptoms for cutaneous forms include skin sores whereas symptoms for visceral form include weight loss, fever, enlarged liver and spleen [156]. Leishmaniasis is one of the common diseases worldwide. Annually about 2 million cases are detected worldwide [157]. Drugs such as sodium stibogluconate and meglumine antimoniate have been used to treat Leishmaniasis. However, these drugs promote development of resistance and severe toxicities such as cardiotoxicity and pancreatitis [158,159]. Due to the resistance developed to the pentavalent antimonial drugs, amphotericin B, miltefosine and paromomycin are used as its alternatives [160,161,162]. However, these drugs produce severe toxicities and are expensive. To avoid these limitations, nanocarriers such as the dendrimers are used to treat leishmaniasis [163].

Dendrimers are a good choice as delivery vehicles due to their biocompatibility and their ability to solubilize the drug and thereby reduce toxicity [164]. Researchers have encapsulated amphotericin B in poly (propyleneimine) (PPI) dendrimers and have noticed that the formulation had a lowered toxicity profile in comparison to the marketed amphotericin B toxicity while remaining active against the parasitic infection observed in the macrophage cell lines and in mice studies. This formulation had an improved ability to target macrophages, unlike amphotericin B alone, and hence has immunomodulatory and antileishmanial activity [165]. Daftarian et al. and Jain et al. have prepared dendritic formulations targeting the Pan-DR binding epitope and PPI containing mannose respectively. Both these dendritic formulations have shown significant improvement in its drug efficacy (around 83%) while reducing the parasitic burden. These formulations have also shown reduced toxicity towards human erythrocytes and macrophages [166,167].

#### 4.3.3. Toxoplasmosis

Toxoplasmosis is caused by the parasite *Toxoplasma gondii,* which infects roughly an estimated two billion people worldwide annually, which causes both morbidity and mortality [168]. Drugs such as pyrimethamine and sulfadoxine are used in the treatment of toxoplasmosis that led to potential toxicities and hypersensitivity. The problem with the traditional forms of therapy is the drugs fail to pass the membranes of the host cells to reach the bradyzoites of *Toxoplasma gondii* [169,170]. Strategy developed for the treatment should surpass the membranes of tachyzoite, bradyzoite and the parasitophorous vacuole. Dendrimers such as the transductive peptide dendrimers can act as an efficient drug delivery tool to deliver the drugs across the several membranes of tachyzoite and encysted bradyzoite, effectively increasing the efficacy of the drug and its toxicity to the parasite [170,171].

Lai and others have prepared conjugates of transductive peptide with phosphorodiamidate morpholino oligomers (PPMO), which resulted in reduced transfection of *Toxoplasma gondii*’s fluorescence, luminescence and reduced tachyzoite replication. In vitro results also translated into in vivo lowering the number of viable parasites after administration [172]. Groups including Prieto et al. have prepared cationic and anionic based dendrimers containing sulfadoxine. In vitro cytotoxicity experiments showed concentrations of these dendrimers from 0.03 to 33 nM of sulfadoxine. Anionic dendrimers showed higher cytotoxicity at higher concentrations while cationic dendrimers have shown increased toxicity at lower concentrations. It can be concluded that lower doses of sulfadoxine in cationic dendrimers act as a good drug delivery tool with significant antitoxoplasmic effect. The antiparasitic effect of dendrimers can be attributed to both surficial activity and endosmolytic effect [173].

## 5. Problems in Using Dendrimers

Despite their significant use in the therapies and drug targeting, dendrimers pose considerable drawbacks as well (Table 3). Due to their cationic surface charge, dendrimers may have toxicity issues such as cytotoxicity and hematological toxicity [174,175,176]. The small size of the dendrimers makes it easy for them to interact with cellular components including nucleus, plasma membrane and endosomes. The toxicity of the dendrimer also depends on the generation of the dendrimer. For example, higher generations of peptide dendrimer have a tendency to interact with the negatively charged cell surface [177,178,179]. Hemolytic toxicity is a common type of toxicity within poly(amido)-amine (PAMAM) dendrimers. This happens due to the interaction of free amino group with the membrane of red blood cells (RBCs). Hemolytic toxicity was observed with PAMAM dendrimer and was further confirmed by mixing dendrimer with the RBC suspension and incubating and then centrifuged at 3000 rpm and analyzing the supernatant at 540 nm spectrophotometrically. Several research groups have concluded that up to 18% hemolysis occurs with generation 4 PAMAM [180,181]. The generation of the dendrimer and the concentration of the dendrimer is proportional to the hemolysis. PAMAM dendrimer could cause hemolysis at a concentration of as low as 1 mg/mL [182].

Many researchers have studied the effects of cationic molecules on the cell membranes. These interactions of the cationic molecules with the cell membranes could cause cell lysis by forming pores in the membrane that are of nanoscale range [183,184,185]. Groups have studied the permeability of the dendrimers in vitro in Madin-Darby Canine Kidney (MDCK) cell lines. The order of permeability of different generation of dendrimers is G4 >> G1 > G3 > G2. This confirmed that the size of the dendrimer and the positive charge of the amino groups could affect the transepithelial transportation of dendrimers significantly [186]. Mecke et al. and Hong et al. have shown how PAMAM dendrimers cause cell lysis using dimrystoylphosphatidylcholine (DMPC) lipid bilayers. G7 PAMAM dendrimers created nanoscale holes in the range of 15–40 nm, whereas G5 dendrimers did not create new nanoscale holes but exacerbated the damage in the lipid bilayers. The cationic charge of the PAMAM dendrimers caused the electrostatic interaction of the dendrimers with the cell surface and taking parts of the lipid bilayer, causing nanoscale holes leading to cell lysis [187]. To overcome these toxicity drawbacks, dendrimers the amine terminal groups were PEGylated, carboxylated or acetylated to lower the toxicity and decrease cell permeability [182,188,189]. The length of the PEG chains conjugated on the dendrimers dictate the cellular interactions, which are surface charge-dependent. Surface functionalization happened to be the best solution to overcome the toxicity issues of dendrimers [190,191]. Recently, it was shown that the use of G4 mixed-surface dendrimers, which has 90% of their surface covered with hydroxyl groups and the other 10% with amine groups, can mitigate the problems caused by traditional PAMAM dendrimers. Use of these dendrimers covered with hydroxyl groups can not only help to reduce the cytotoxicity caused by the PAMAM dendrimers but they are easy to fluorescently label and target neuron and glial cells [192]. Their capability for targeting neuron and glial cells can help them overcome problems caused by traditional PAMAM dendrimers, which is their inability to cross the blood brain barrier (BBB). Some research groups have suggested limiting the surface functionalization to a minimum to maintain the benefits of these dendrimers including their purity and safety. Minimizing the surface functionalization also helps prevent unpredictable ADME profiles caused by surface functionalization while retaining their original properties [193,194].

Another important drawback of dendrimer system is the rapid renal clearance associated with its smaller size (G2 to G4). This property of rapid renal clearance makes it difficult to increase the residence time in the body, thus making it difficult for sustained drug delivery. Larger dendrimers are cleared by the reticuloendothelial system (RES) [195]. One of the ways to improve the pharmacokinetic profile of the dendrimers has been to surface functionalize using PEG. Conjugation of the PEG chain on to the dendrimer surface can create a stealth layer that makes it tough for the dendrimer to be recognized by RES. Moreover, functionalization with PEG also increases the molecular weight, making it more difficult to be cleared via the renal route to increase circulation time. Chain length of the PEG to be surface modified can be selected based on the pharmacokinetic profile that is desired [196,197,198]. However, the use of long chain PEGs for longer circulation has raised a few immunological concerns with anti-peg antibodies. These are found in healthy subjects, which could have been a result of the use of PEG containing cosmetics and foods [199,200].

The iterative synthetic process used to synthesize dendrimers is a slow, complex and costly process as it requires long reaction time with multiple synthetic steps. However, with a few advancements in the past few years this process was expedited and simplified. Orthogonal coupling strategy and click chemistry are some of the important ways this synthesis process can be expedited. Orthogonal coupling strategy can be used to decrease the synthesis steps while click chemistry can be used for high yields with non-toxic byproducts [201,202]. G2 and G3 triazole dendrimers were used as pure solids using Cu(I) catalyzed click chemistry with sodium chloride as the byproduct [44]. These improvements in the dendrimer synthesis process can improve the scalability and reproducibility of the dendrimer-based materials and the surface modification can decrease the toxicities and can alter the pharmacokinetic profiles in a positive way. Although these drawbacks have hindered the clinical translation of dendrimers so far, improvements in clinical translatability of the dendrimer-based nanoparticles have a promising future.

**Table 3:** Problems of dendrimers and ways to overcome the undesirable effects.

Problems/Undesirable Effects	Methods to Resolve Undesirable Effects	References
Interaction with cell membranes due to their cationic charge causing cell lysis	PEGylation, acetylation or carboxylation of the terminal groups Use of mixed-surface dendrimers, which have fewer numbers of amines on the surface and higher numbers of hydroxyl groups on the surface (–OH)	[182,183,188,203,204]
Hematological toxicity due to its cationic charge	PEGylation, acetylation or carboxylation of the terminal groups	[180,181]
Rapid Renal Clearance	PEGylation to increase circulation time	[195,196,197,198]
Slow, complex and costly synthetic process	Orthogonal coupling and click chemistry are ways to resolve this slow, complex and costly process	[201,202]
Inability to penetrate the blood brain barrier (BBB)	Use of mixed-surface dendrimers have proven to be effective in delivering the cargo across BBB	[194]

## 6. Use of Dendrimers as Diagnostics

Infectious diseases, especially viruses and bacteria caused diseases, are directly related to the occurrence and development of various kinds of cancers. Hepatitis B virus (HBV) is related to primary liver cancer [205]. “Hepatitis-cirrhosis-cancer” represents the “three steps” of viral hepatitis developing into liver cancer. According to a survey, about 70–80% of liver cancer developed from hepatitis B. Human papillomavirus (HPV) is associated with cervical cancer [206]. Women infected with HPV are more likely to get cervical cancer than those without HPV infection. Epstein Barr virus (EBV) is related to nasopharyngeal carcinoma and lymphoma [207]. EBV is a kind of herpesvirus that exists all over the world. It is not only related to nasopharyngeal carcinoma and lymphatic carcinoma, but also possesses connections to gastric cancer, lung cancer, breast cancer and cervical cancer [208,209]. *Helicobacter pylori* (Hp) is related to the occurrence of gastric cancer [210]. This kind of bacteria will enter the stomach with food and destroy the gastric mucosa. When the gastric mucosa is repeatedly damaged and repaired for a long time, it may mutate into a tumor. The long-term imbalance of intestinal microbiota may lead to colorectal cancer [211]. A large number of studies have found that there is a considerable number of cancer promoting bacteria in the human intestinal tract, including *Bacteroides fragilis, Enterococcus faecalis*, Bacteroides, Firmicutes and Lactococcus and fusobacteria. The structure of the intestinal microbiota and its metabolites can affect the susceptibility of the body to diseases, and may even directly induce colorectal cancer and other pathological states. Since there is a deep connection between infectious diseases and cancer, and the direct application of dendrimers in the diagnosis of infectious diseases is rarely reported, we reviewed the application of dendrimers in the diagnosis of cancer caused by infectious diseases.

By linking to biocompatible NPs, proteins or polymers by stimulus-responsive chemical bonds, lower-generation dendrimers temporarily produce hybrid materials with relatively high charge density that are capable of efficient, nontoxic gene delivery while enabling the NPs to function as diagnostic agents. The introduction of inorganic NPs (such as Au, iron oxide and quantum dots) endows these hybrid gene carries with new functions. The unique cavity structure of dendrimers can encapsulate or stabilize metal NPs for cancer imaging. For example the use of dendrimers as the template to encapsulate Au NPs for computed tomography (CT) imaging of cancers, the use of dendrimers surface-modified with Gd(III) ligand chelator to encapsulate Au NPs for CT/magnetic resonance (MR) bimodal imaging [212] and the use of dendrimers surface-chelated with isotope 99mTc or Mn(II)/99mTc to encapsulate Au NPs for single-photon emission CT (SPECT)/CT or SPECT/MR bimodal imaging of cancers [213,214,215,216]. In addition, owing to their hydrophobic internal environment, dendrimers can effectively encapsulate anticancer drug molecules and, with the surface modified with a targeting agent, can be used for targeted diagnosis and therapy of cancers.

### 6.1. Research Progress and Applications of Dendrimers in SPECT Imaging

SPECT is an imaging technology that obtains the morphology, position and functionality of tissues and organs by collecting and displaying the distribution density and flow of radioisotopes in the human body. More than just the imaging of anatomical structures, SPECT can detect functional changes of organs or systems and effectively support disease diagnosis. However, the clinical application of this technology is limited by the less-than-desirable properties of the available radioisotopes (such as short half-lives, lack of tissue-specificity and other disadvantages).

Owing to their good physiochemical properties, dendrimers can be used as nanoplatforms to carry radioisotopes for SPECT imaging. Xu et al. [217] reacted a partially acetylated generation-5 PAMAM (G5-Ac) with biotin and 2-(*p*-isothiocyanatobenzyl)-6-methyl-diethylenetria minepentaacetic acid (1B4M-DTPA) successively. The resulting complex, Bt-G5-Ac-1B4M, was subsequently covalently bonded with avidin. The resulting system, Av-G5-Ac-1B4M, was radiolabeled with 99mTc to obtain a nanomaterial, Av-G5-Ac-1B4M-99mTc. In vitro cellular uptake tests confirmed that the avidin-bonded carrier was remarkably effective in targeting HeLa cells.

Zhang et al. [218] surface-modified a partially acetylated G5 dendrimer (G5.NH_2_) with folic acid (FA)-containing polyethylene glycol (PEG) and diethylenetriaminepentaacetic acid (DTPA) chelator and subsequently radiolabeled the resulting conjugate with 99mTc. The resulting imaging agent, 99mTc-G5-Ac-peg FA-DTPA, was compared with a nanomaterial not modified with PEG, 99mTc-G5-Ac-FA-DTPA and a nanomaterial not modified with PEG-FA, 99mTc-G5-Ac-DTPA. In vivo micro-SPECT imaging results showed that the nanomaterials accumulated mainly in the kidney, liver and tumors, without marked accumulation in other tissues and organs. In addition, mouse tumor models treated with 99mTc-G5-Ac-peg FA-DTPA yielded stronger SPECT imaging signals than those treated with 99mTc-G5-Ac-FA-DTPA or 99mTc-G5-Ac-DTPA. Therefore, 99mTc-G5-Ac-peg FA-DTPA was more effective in targeted SPECT imaging of tumors.

### 6.2. Application of Dendrimers in CT Imaging

X-ray CT is based on the principle that tissues and organs have different compositions and densities and thus attenuate the passing X-ray beam at different degrees. In other words, CT images are obtained by measuring the intensity of the passed X-ray beam. CT imaging agents are used to increase the contrast between tissues/organs. A stronger contrast leads to clearer images. Thus, more accurate diagnostic information can be obtained [219]. Recently this method is frequently used as a helpful tool for COVID-19 disease diagnosis. In the case of emergency disease control, chest CT provides a rapid and effective method to early recognize suspicious cases [220], especially for patients with COVID-like symptoms with a negative result for RT-PCR [221].

Yordanov et al. [222] used a generation-4 (G4) PAMAM dendrimer as the carrier and bonded an iodine compound, 3-*N*-[(*N*′,*N*′-dimethylaminoacetyl)amino]-α-ethyl-2,4,6-triiodobenzenepropanoic acid (DMAA-IPA), to the surface to obtain a iodinated imaging agent G4-(DMAA-IPA), which had a high iodine content of 33.1%. This was the first successful attempt to bind a small-molecule iodine compound and a dendrimer for CT imaging.

In addition to iodine, many other heavy-metal NPs have received increasing attention in medicine, particularly imaging diagnostics of diseases, due to their excellent properties in optics and quantum size. Because of their high X-ray attenuation coefficient, Au NPs are a good CT imaging agent. Dendrimers loaded with CT imaging and chemotherapeutic agents can realize effective targeted CT imaging and chemotherapy of tumors. Functionalized and modified dendrimer platforms are capable of precise imaging and efficient treatment of tumors, providing solutions for combined monitoring and early treatment of cancers.

Using a G5 PAMAM dendrimer as the carrier, Zhu et al. [223] synthesized dendrimer-entrapped Au nanoparticles (Au DENPs) surface-modified with anticancer α-tocopheryl succinate (α-TOS). The resulting theranostic nanomaterial α-TOS-Au DENPs had an average particle size of 3.3 nm and 9.8 α-TOS molecules per dendrimer and were fairly stable under different pH, temperature and solvent conditions. To thoroughly investigate the anticancer mechanism of the α-TOS-Au DENPs above, Zhu et al. [224] synthesized an Arg-Gly-Asp (RGD)-targeting α-TOS-Au DENPs system. Compared with pure α-TOS, the RGD-targeting α-TOS-Au DENPs induced tumor cells to generate a higher level of reactive oxygen species, thereby enhancing cancer cell apoptosis. Zheng et al. [225] synthesized AU DENPs using a G5 PAMAM functionalized with FA and methotrexate (MTSX) as the template. The target specificity of FA enabled the theranostic nano agent to bind specifically with cancer cells overexpressing FA receptors, thereby increasing the uptake of the nano agent into these cancer cells. In addition, the therapeutic effect of MTX enabled specific inhibition of the growth of these cancer cells. Moreover, the Au NPs functioned as an agent for CT imaging.

### 6.3. Application of Dendrimers in MR Imaging

MR uses externally applied magnetic field gradients to detect electromagnetic signals from the human body, which are then computed to reconstruct anatomical information [226]. MR imaging is similar to other tomographic imaging techniques. All these techniques obtain the spatial distribution of a physical quantity. However, MR imaging can obtain the tomographic and three-dimensional images in any direction. However, to improve the detection sensitivity, image definition and diagnostic accuracy of MR imaging, the assistance of imaging agents is necessary.

Small-molecule metal agents commonly used at present for MR imaging have the following disadvantages that limit their clinical application: (1) short residence in and quick removal from the blood circulation system; (2) lack of targeting capability; (3) small signal-to-noise ratio. Dendrimers have an exceptional macromolecular structure that contains unique internal cavities and many modifiable surface functional groups. Therefore, MR imaging agents can be bound to dendrimers. In addition, MR imaging nano agents with stable properties, good biocompatibility, high relaxivity, long blood circulation time and targeting capability can be obtained through functional modification of the dendrimer surface [227,228,229].

Mohamadi et al. [230] synthesized a new MR imaging agent by attaching Gd molecules to a dendrimer and evaluated the hepatic uptake and cytotoxicity of the synthesized agent by performing in vitro and in vivo imaging tests. The results showed that the binding of Gd and the dendrimer produced a safer, more efficient imaging agent.

Mustafa et al. [231] proposed a facile approach for synthesizing dendrimer-functionalized, Gd-loaded LAPONITE^®^ (LAP) nano disks for in vitro and in vivo T1-weighted MR imaging. Cell viability assays showed that the synthesized LM-G2-DTPA (Gd) nanocomposite was non-cytotoxic in the given concentration range, had a high r1 relaxivity and was an efficient contrast agent for T1-weighted MR imaging of cancer cells in vitro and animal organs/tumor models in vivo.

Gadolinium-containing G4 dendrimer was also used as a biomarker for sepsis-induced acute renal failure (ARF). This approach helps to detect renal injury at an early stage, and provide information about the cause, response to therapy and prognosis [232]. To replace gadolinium dendrimers as contrast agents, G0- and G1-OEG-PROXYL radical dendrimers were created as a safer option. These dendrimers can be considered potential candidates as alternatives to Gd-based contrast agents currently used in MRI applications such as in follow-ups of infectious diseases [233].

The PAMAM-G8 dendrimers were also used as a MR imaging contrast agents in a mouse model. The purpose of this study was to visualize the lymphatic flow and lymph nodes to distinguish between the dilation of lymphatic vessels, proliferative or neoplastic lymph node swellings and changes in lymph nodes caused by infection/inflammation [234].

Yang et al. [235] synthesized carboxyl (COOH)-modified ultrasmall Fe_3_O_4_ NPs (Fe_3_O_4_-COOH NPs), which were covalently conjugated with RGD-modified G5.NH_2_ (G5.NH_2_-RGD), followed by acetylation of the remaining dendrimer terminal amines. The resulting T2 imaging agent, G5.NHAc-RGD-Fe_3_O_4_ NPs, specifically bound with integrin-overexpression C6 glioma cells. The results of cell viability assays, cell morphological observations and hemolysis assays showed that the synthesized G5.NHAc-RGD-Fe_3_O_4_ NPs had good biocompatibility and hemocompatibility. In vivo tumor MR imaging tests showed that the RGD-mediated targeting increased the uptake of G5.NHAc-RGD-Fe_3_O_4_ NPs by tumor cells. These studies provided ideas for effective early diagnosis of cancers and showed the potential of these nanoplatforms for targeted MR imaging of various cancers.

A similar approach was used to prepare dendronized magnetic nanoparticles. NP’s containing Fe^2+^/Fe^3+^ ions were bound to dendrons functionalized with carboxyl groups at the periphery. These carbosilane dendrimers can capture different HIV-1 isolates, due to electrostatic interactions between carboxylate groups and HIV-1 antibodies of the samples. The exact mechanism by which the dendrimers bind to HIV-1 is still not clear, but this research provides a new approach for faster HIV-1 detection, which reduces the waiting of 2–4 weeks required by current screening techniques [236].

### 6.4. Application of PAMAM Dendrimers in CT/MR Bimodal Imaging

Significant research on contrast agents for RT/MI bimodal imaging has been conducted. For example, with Au NPs functionalized with Gd ions [237,238], core–shell NPs [239,240,241] and dendrimer-based bimodal nanomaterials [212,242,243,244,245,246].

Cai et al. [242] used dendrimer based Fe_3_O_4_/Au composite NPs for targeted CT/MR bimodal imaging. Fe_3_O_4_ NPs were assembled with multilayers of poly (γ-glutamic acid) (PGA)/poly(l-lysine)/PGA/folic acid (FA)-modified Au DNEPs using a layer-by-layer self-assembly technique. The synthesized nanomaterial exhibited a relatively high R2 relaxivity, good X-ray attenuation properties and good cytocompatibility and hemocompatibility in the tested concentration range. With the FA-mediated targeting, the NPs were specifically uptaken by cancer cells overexpressing FA receptors and were an efficient probe for targeted CT/MR bimodal imaging of a xenografted tumor model.

Chen et al. [243] synthesized Au DENPs loaded with Gd chelator/Gd(III) complexes and surface-modified with the targeting agent Arg-Gly-Asp-Phe-Lys (mpa) (RGD peptide) for targeted CT/MR bimodal imaging of tumors. The resulting multifunctional Au DENPs (symbolized as Gd-Au DENPs-RGD) were characterized using different techniques. The results showed that the multifunctional DENPs had an Au core size of 3.8 nm, had good water-dispersibility, were stable over a different pH range (5–8) and temperature range (4–50 °C) conditions and had a low cytotoxicity at an Au concentration up to 100 μM. In addition, the synthesized Gd-Au DENPs-RGD had good X-ray attenuation properties and a high r1 relaxivity and was an efficient nanoprobe for targeted CT/MR bimodal imaging of a xenografted small tumor model overexpressing αvβ3 integrin.

Wen et al. [246] used Gd-loaded Au DENPs for CT/MR bimodal imaging. The Au DENPs were synthesized using G5.NH_2_ surface-modified with Gd chelator and PEG monomethyl ether as the platform, followed by chelation of Gd(III) and acetylation of the dendrimer surface. The synthesized contrast agent (Gd-Au DENPs) was efficient for CT/MR bimodal imaging of the heart, liver, kidney and bladder of rat and mouse models. In addition, an in vivo biodistribution study showed that the Gd-Au DENPs had an extended blood circulation time that was cleared from the major organs in 24 h.

Functionalized and modified dendrimer platforms are capable of precise imaging and efficient treatment of infectious related tumors, providing solutions for combined monitoring and early treatment of cancers. However, currently available dendrimer-based theranostic agents have some deficiencies (Table 4). For example, the loading of hydrophobic agent affects the stability and water-solubility of Au DENPs, and these Au DENPs are prone to precipitation when stored for a long time. Therefore, synthesis of functionalized dendrimers that provide diagnostic and therapeutical effect while maintaining good stability and water-solubility is at the present a pressing problem and a direction for future research of dendrimer-based theranostic agents.

### 6.5. Application of Dendrimers in ELISA-Like Assays

**Figure 6:**
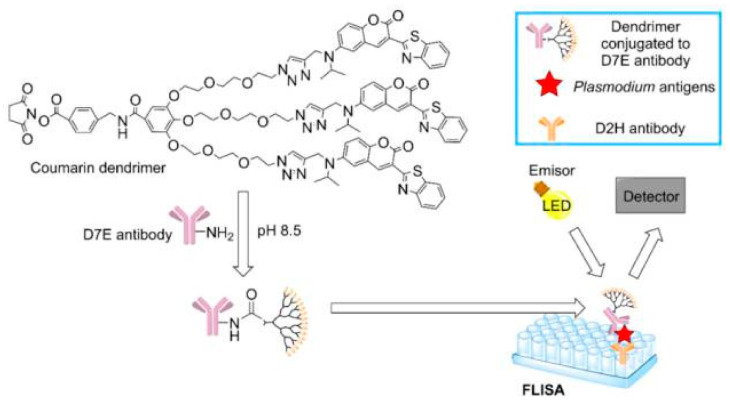
Dendrimer-based assay to detect malaria. Schematic mode of the detection by fluorescence-linked immunosorbent assay (FLISA) [255].

Due to some limitation of conventional infectious disease diagnostics, including imaging, polymerase chain reaction (PCR) and enzyme-linked immunosorbent assay (ELISA), new diagnostic methods have been in demand. In South Korea, a dendrimer-based assay to detect malaria has been approved as a rapid and cheap diagnostic tool. The assay is based on fluorescence as shown in Figure 6 [255].

The created coumarin-derived dendrimer-based fluorescence-linked immunosorbent assay (FLISA) can detect two malaria-specific antigens: histidine-rich protein II (HRP2) and lactate dehydrogenase (LDH). The advantage of this method is that it provides higher sensitivity than traditional ELISA, which can be useful to detect asymptomatic cases [256].

Another dendrimer assay that detects *Schistosoma* circulating anodic antigen (CAA) is comprised of magnetic particles coated with G4-PAMAM-NH2. The principle of this assay involves the electrostatic interactions between the negatively-charged CAA biomarker and positively charged poly(amidoamine) (PAMAM) dendrimers modified with magnetic nanoparticles. The advantage of this method is that enables concentration of the sample, and thus becomes less time-consuming and waives the requirement of significant laboratory infrastructure with detection of the CAA antigen of 200-fold on a lateral flow assay when compared to currently used assays [257].

## 7. Conclusions

Dendrimers are polymers with a very interesting structure and unique properties. The article presents these compounds as molecules for treatment and prevention for infectious diseases, but it should be noted that they perform well in medical sciences, bioscience, nanotechnology and modern industrial technologies. However, many potential applications of dendrimers are still in the early phases of intensive research, including their use in medicine. It is not well known what the metabolism of these polymers is, how they were taken, for example, in the form of oral drugs, and what impact the metabolites of dendrimers may have on cells, tissues and organs. Research on dendrimers is ongoing and very intensive. Although clinical translation is difficult so far, improved manufacturing technologies and surface modifications of dendrimers may make the clinical translatability easy. These dynamically developing innovative branches of nanotechnology and nanomedicine constitute a wide-open field for the search for new applications of spherical polymers, such as dendrimers.

## Figures and Tables

**Table 1:** Advantages and examples of dendrimer usage in medicine.

Biomedical Applications of Dendrimers	Advantages of Using Dendrimers	Examples
Contrasting factor for magnetic resonance imaging	Optimal presence time in the body No risk of deposition of gadolinium compounds. High-quality imaging	Gadomer 17.
		Contrast agent containing chelates of gadolinium ions based on lysine dendrimers [53]
Drug carrier: - molecules locked in the nodes between the arms - molecules attached to dendrimers surface groups	Increased solubility of hydrophobic drugs in the aqueous environment of body fluids [54] Increased the permeability of cell membranes to drugs Protection of drugs during transport in the body Controlled, sustained drug release, to lower toxicity To lower doses of the drug Minimization of adverse drug effects Simultaneous attach to one dendrimer molecule to various medicinal substances To “address” the carrier dendrimer molecule for drug-targeted therapy [54]	Niclosamide.
		An antiparasitic drug, almost insoluble in water—after encapsulation in PAMAM dendrimers. Solubility increased. Enables the drug release to be controlled [55]
		Fluorouracil (5FU). An anticancer drug, combined with a different generation of PAMAM dendrimer inhibited E6 and E7 oncogene activity. Showed slower drug release, lower toxicity and greater tumor accumulation [56]
		Ketoprofen (a non-steroidal anti-inflammatory drug). A conjugate with the PAMAM dendrimer was better soluble in water, blood concentration was increased, and action was prolonged [57]
		G4 PAMAM with tetracycline [58]
		G4 PAMAM dendrimers linked with Trastuzumab (HER2 antibody) for breast tumor targeted delivery [59]
Gene therapy (non-viral gene transporters)	High efficiency nucleic acids transport Protection against degradation Stability of complex over a wide pH range Codelivery with other molecules	SuperFect^®^ (Qiagen, Hilden, Germany). Acommercially available transfection PAMAM agent based on dendrimers [60]
		G3 PPI dendrimer with 1,4-diaminobutane as core combined with plasmid DNA [30]
		siRNA and doxorubicin codelivery system for the treatment of multidrug resistance cancers [61]
Dendrimer vaccines	Binding multiple copies of the antigen to increase immunogenicity of the vaccine Omission of protein carriers that are potential allergenic factors	Vaccine against malaria. Built of MAP (multiple antigenic peptide) dendrimers [62]
		Vaccine against Ebola virus. A DNA containing vaccine based on 4 polyamidoamine dendrimers (PAMAM G4) [63]
Antiviral and antibacterial drugs with a dendrimeric structure	Block receptors on the surface of the virus Prevent viruses or bacteria from attaching to mammals cells by covering them Damage the anionic membrane of the bacterial cell, inducing its lysis	Sialodendrimers. Inhibition of hemagglutination of human erythrocytes caused by the influenza virus [64]
		VivaGel^®^ (Starpharma Ltd., Melbourne, Australia). Based on polylysine (G4) dendrimers. Protection against HIV infection [65]
Treatment of neurodegenerative diseases (Alzheimer’s, Parkinson’s, prion diseases)	Prevent formation of harmful amyloid deposits Break down existing fibrillar protein aggregates	PAMAM G3, G4 and G5 dendrimers. Inhibition of the formation of amyloid deposits. Characterized by the ability to degrade existing aggregates [66]
Anti-inflammatory molecules	Inhibit the synthesis of proteins secreted during the induction of an inflammatory reaction Reduce inflammatory response Enhanced water solubility	35% PEGylated G4 PAMAM and 45% PEGylated G6 PAMAM dendrimers—increased uptake. Negligible acute or chronic histotoxicity [67]
		Phosphor dendrimers coated with bisphosphonate residues (ABP) as above.They enhance the proliferation of NK cells.
		PAMAM dendrimers show anti-inflammatory activity in vivo using models representing acute and chronic inflammatory response [68]

**Table 2:** Antiviral application of dendrimers.

Virus	Dendrimer Type	Payload	References
Hepatitis C	Carbosilane dendrimers	Sofosbuvir	[124]
	PETIM	siRNA	[125]
HPV (Cervical cancer)	Peptide dendrimers	siRNA	[126]
		Doxorubicin	[127]
FMDV (Foot-and-mouth disease virus)	Peptide dendrimer	Cyclic disulfide epitope	[128]
RVS (Respiratory Syncytial Virus)	RFI-641		[129]

**Table 4:** Advantages and limitations of dendrimers for imaging purposes.

Imaging Technique	Advantages	Limitations
SPECT	Highly branched architecture, adequate spatial cavities and tremendous functional terminals [247], prolonged circulation time, ease of conjugation with drugs and active targeting agents [248,249,250], low cytotoxicity and efficient cell entry capability [247]	The apparent accumulation in the liver and spleen [247].High cost of their complex preparation methods that usually involve multistep syntheses and their toxicity [245,251,252].
CT	Biocompatibility [253], high contrast enhancement in the blood-pool and effectively extended their blood half-lives, preventing undesirable long-term accumulation in vivo and attaining reproducibility for their syntheses and properties [252].	
MR	Rapid blood clearance and lower liver uptake [251], high longitudinal relaxivity and consistent contrast enhancement [254]	
CT/MR	Biocompatibility, monodispersity and in vivo stability, particle size controllability, realization of multimodal imaging [245] and high payload [243].	
